# Supplementary material for: Seroepidemiology of Borrelia burgdorferi s.l. among German National Cohort (NAKO) Participants, Hanover
Source: Microorganisms. 2022 Nov 17;10(11):2286. doi: 10.3390/microorganisms10112286 (PMC9694946; doi:10.3390/microorganisms10112286)
Supplement: Supplementary file 1 [file microorganisms-10-02286-s001.zip › microorganisms-2015134-supplementary.pdf]

Supplementary Material

Article: Seroepidemiology of *Borrelia burgdorferi* s.l. among German National Cohort (NAKO) Participants, Hanover

Authors: Max J. Hassenstein, Irina Janzen, Gérard Krause, Manuela Harries, Vanessa Melhorn,
Tobias Kerrinnes, Yvonne Kemmling, Stefanie Castell

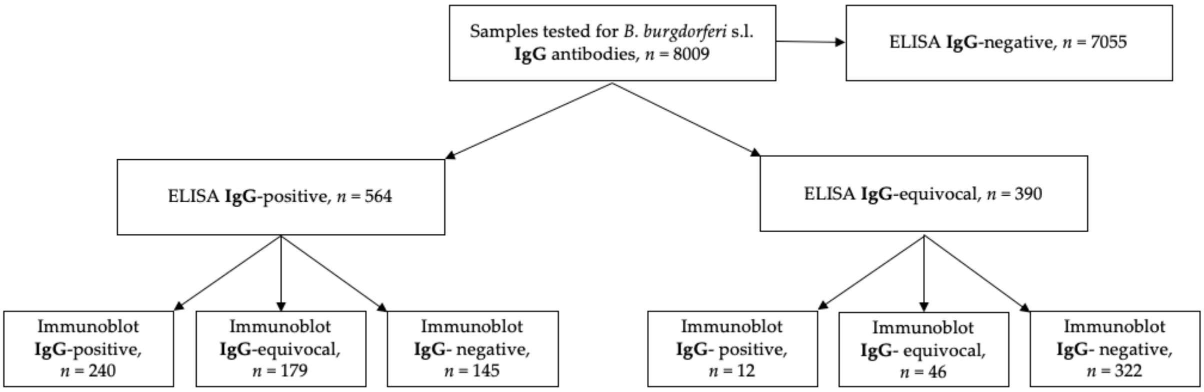

Figure S1. Flowchart from two-tier sample testing for IgG antibodies against *B. burgdorferi* s.l. IgG = Immunoglobulin G.

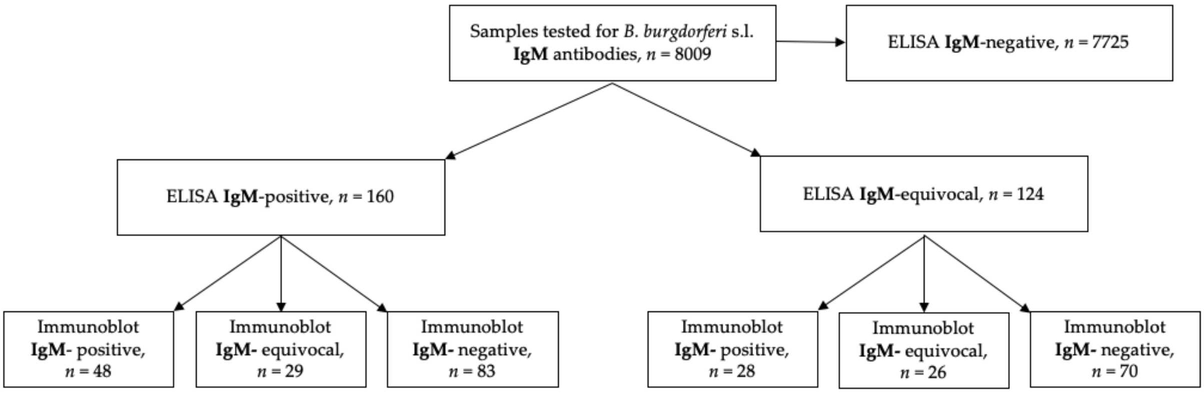

Figure S2. Flowchart from two-tier sample testing for IgM antibodies against *B. burgdorferi* s.l. IgM = Immunoglobulin M.

Table S1. Implementation and comparison of force of infection (FOI) models.

Model	GLM FOI Formula [1]	GLM Implementation in R Programming Language [1,2]	AIC
Muench [1,2]	$\eta(a) = \log(\beta) + \log(a)$	glm(cbind(Positive,Total-Positive) ~ 1, offset=log(Age), family = binomial(link = "cloglog"))	200.55
Griffiths [3]	$\eta(a) = \beta_1 a + \beta_2 a^2$	glm(cbind(Total-,Positive) ~ −1 + Age + I(Age^2), family = binomial(link = "log"))	202.54
Grenfell and Anderson [4]	$\eta'(a) = \beta_1 + 2\beta_2 a + 3\beta_3 a^2$	glm(cbind(Total-,Positive) ~ −1 +Age + I(Age^2) + I(Age^3), family = binomial(link = "log"))	192.39

GLM: generalized linear model; AIC: Akaike information criterion; η: linear predictor; a: age; β: transmission parameter; [1] as suggested by Hens et al. [5]; [2] The glm() call fits a generalized linear model; Positive: vector containing seropositive counts; Total: vector containing the sum of seropositives and seronegatives; Age: vector containing the participants' age in years.

Table S2. Odds Ratios for IgG/IgM seropositivity from logistic regression.

Independent Variables	Odds Ratio for IgG Seropositivity [1] (95% CI)	*p*	Odds Ratio for IgM Seropositivity [1] (95% CI)	*p*
**Age, 10-year increments**	1.26 (1.13–1.42)	**<0.001**	1.07 (0.88–1.30)	0.512
**Sex**				
Female	Ref.	Ref.	Ref.	Ref.
Male	2.58 (1.94–3.46)	**<0.001**	1.24 (0.78–2.00)	0.362
**Migration background [1]**				
No	Ref.	Ref.	Ref.	Ref.
Yes	0.71 (0.48–1.02)	0.071	1.02 (0.56–1.75)	0.947
**Education [2]**				
Ongoing	1.03 (0.24–2.97)	0.968	2.49 (0.66–7.67)	0.138
Low	0.39 (0.06–1.25)	0.188	1.62 (0.38–4.84)	0.440
Medium	Ref.	Ref.	Ref.	Ref.
High	1.22 (0.92–1.63)	0.179	1.39 (0.83–2.39)	0.223
**Net equivalent monthly income, 100-EUR increments**	1.00 (0.99–1.00)	0.325	0.98 (0.96–1.00)	**0.038**
**Body Mass Index [3]**	0.96 (0.93–0.99)	**0.021**	0.95 (0.90–1.01)	0.105
**Depression status (PHQ-9, point increase) [4]**	0.94 (0.90–0.98)	**0.006**	0.99 (0.93–1.05)	0.780
**Smoking status**				
Never	Ref.	Ref.	Ref.	Ref.
Former	1.10 (0.82–1.48)	0.523	1.40 (0.81–2.42)	0.224
Current	1.05 (0.71–1.52)	0.818	1.11 (0.54–2.18)	0.765
Unknown	1.30 (0.80–2.03)	0.274	1.38 (0.59–2.95)	0.421

IgG = Immunoglobulin G; IgM = Immunoglobulin M; Ref = reference; CI = confidence interval; Observations = 8009. We considered a sample as seropositive for *Borrelia burgdorferi* s.l. with positive or equivocal ELISA and subsequent positive immunoblot result (MiQ12) [6]; [1] Migration derived from a minimum set of indicators by Schenk et al. [7]; [2] Education level derived according to ISCED97 [8] ; [3] BMI corresponding to the classification of the International Obesity Task Force [9] ;[4] Depression status obtained from the 9-question Patient Health Questionnaire (PHQ-9) [10]

Table S3. Estimated mean force of infection (FOI) for IgG seropositivity against *B. burgdorferi* s.l.

Age (years)	FOI Estimation Muench [1]	Griffiths [2]	Grenfell and Anderson [3]
20–24	0.000637	0.000634	0.0000656
25–29	0.000637	0.000637	−0.000153
30–34	0.000637	0.000641	−0.000230
35–39	0.000637	0.000644	−0.000167
40–44	0.000637	0.000647	0.0000375
45–49	0.000637	0.000651	0.000383
50–54	0.000637	0.000654	0.000869
55–59	0.000637	0.000657	0.00150
60–64	0.000637	0.000661	0.00226
65–69	0.000637	0.000664	0.00317

The FOI represents the average annual change in the population's seropositivity proportion. IgG = Immunoglobulin G; [1] Muench's model [1,2] assumes constant FOI over age; [2] [3]; [3] [4], Best performing model considering AIC.

References

1. Muench, H. Derivation of Rates from Summation Data by the Catalytic Curve. *Journal of the American Statistical Association* **1934**, *29*, 25, doi:10.2307/2278457.
2. Muench, H. *Catalytic Models in Epidemiology;* Harvard University Press: Cambridge, MA, USA, 1959, ISBN 9780674428928.
3. Griffiths, D.A. A Catalytic Model of Infection for Measles. *Applied Statistics* **1974**, *23*, 330, doi:10.2307/2347126.
4. Grenfell, B.T.; Anderson, R.M. The estimation of age-related rates of infection from case notifications and serological data. *J. Hyg. (Lond)* **1985**, *95*, 419–436, doi:10.1017/s0022172400062859.
5. Hens, N.; Shkedy, Z.; Aerts, M.; Faes, C.; van Damme, P.; Beutels, P. *Modeling Infectious Disease Parameters Based on Serological and Social Contact Data;* Springer New York: New York, NY, 2012, ISBN 978-1-4614-4071-0.
6. *Lyme-Borreliose;* Mauch, H.; Fingerle, V.; Eiffert, H.; Gessner, A.; Göbel, U.; Hofmann, H.; Hunfeld, K.-P.; Krause, A., Eds., 2. Auflage; Urban & Fischer in Elsevier: München, 2017, ISBN 978-3-437-22605-2.
7. Schenk, L.; Bau, A.-M.; Borde, T.; Butler, J.; Lampert, T.; Neuhauser, H.; Razum, O.; Weilandt, C. Mindestindikatorensatz zur Erfassung des Migrationsstatus. Empfehlungen für die epidemiologische Praxis. *Bundesgesundheitsblatt Gesundheitsforschung Gesundheitsschutz* **2006**, *49*, 853–860, doi:10.1007/s00103-006-0018-4.
8. *International standard classification of education ISCED 1997*, English edition. - Re-edition; UNESCO-UIS: [Montreal], 2006, ISBN 92-9189-035-9.
9. Cole, T.J.; Lobstein, T. Extended international (IOTF) body mass index cut-offs for thinness, overweight and obesity. *Pediatr. Obes.* **2012**, *7*, 284–294, doi:10.1111/j.2047-6310.2012.00064.x.
10. Kroenke, K.; Spitzer, R.L. The PHQ-9: A New Depression Diagnostic and Severity Measure. *Psychiatric Annals* **2002**, *32*, 509–515, doi:10.3928/0048-5713-20020901-06.
